# Pterostilbene Targets Hallmarks of Aging in the Gene Expression Landscape in Blood of Healthy Rats

**DOI:** 10.1002/mnfr.202400662

**Published:** 2024-11-19

**Authors:** Marco A. Tello‐Palencia, Tony Yang, Olga Sularz, Louis Erik Demers, Yuexi Ma, Cayla Boycott, Huiying Amelie Zhang, Katarzyna Lubecka‐Gajewska, Sadhri Kumar, Benjamin S. Ramsey, Sandra Torregrosa‐Allen, Bennett D. Elzey, Nadia Atallah Lanman, Keegan Korthauer, Barbara Stefanska

**Affiliations:** ^1^ Department of Statistics Faculty of Science the University of British Columbia Vancouver BC V6T 1Z4 Canada; ^2^ Food, Nutrition and Health Program Faculty of Land and Food Systems the University of British Columbia Vancouver BC V6T 1Z4 Canada; ^3^ Department of Human Nutrition and Dietetics Faculty of Food Technology University of Agriculture in Krakow Krakow 31‐120 Poland; ^4^ Department of Parasitology McGill University Montreal QC H9X 3V9 Canada; ^5^ Department of Biomedical Chemistry Medical University of Lodz Lodz 92‐215 Poland; ^6^ Purdue University Institute for Cancer Research Purdue University West Lafayette IN 47906 USA; ^7^ Department of Comparative Pathobiology College of Veterinary Medicine Purdue University West Lafayette IN 47907 USA; ^8^ Centre for Molecular Medicine and Therapeutics BC Children's Hospital Research Institute Vancouver BC V6H 0B3 Canada

**Keywords:** aging, blood, gene expression, polyphenols, transcriptomics

## Abstract

**Scope:**

Polyphenols from the phytoestrogen group, including pterostilbene (PTS), are known for their antioxidant, anti‐inflammatory, and anti‐cancer effects. In recent reports, phytoestrogens attenuate age‐related diseases; however, their pro‐longevity effects in healthy models in mammals remain unknown. As longevity research demonstrates age‐related transcriptomic signatures in human blood, the current study hypothesizes that phytoestrogen‐supplemented diet may induce changes in gene expression that ultimately confer pro‐longevity benefits.

**Methods and results:**

In the present study, RNA sequencing is conducted to determine transcriptome‐wide changes in gene expression in whole blood of healthy rats consuming diets supplemented with phytoestrogens. Ortholog cell deconvolution is applied to analyze the omics data. The study discovered that PTS leads to changes in the gene expression landscape and PTS‐target genes are associated with functions counteracting hallmarks of aging, including genomic instability, epigenetic alterations, compromised autophagy, mitochondrial dysfunction, deregulated nutrient sensing, altered intercellular interaction, and loss of proteostasis. These functions bridge together under anti‐inflammatory effects through multiple pathways, including immunometabolism, where changes in cellular metabolism (e.g., ribosome biogenesis) impact the immune system.

**Conclusion:**

The findings provide a rationale for pre‐clinical and clinical longevity studies and encourage investigations on PTS in maintaining cellular homeostasis, decelerating the process of aging, and improving conditions with chronic inflammation.

## Graphical abstract

The study characterizes transcriptomic patterns in blood of healthy rats consuming diets supplemented with polyphenols. Supplementation with pterostilbene (PTS), a dietary bioactive compound from a group of phytoestrogens, resulted in gene expression changes that are functionally linked to hallmarks of aging. It suggests PTS action towards reduced inflammation and maintenance of homeostasis in the immune functions and cellular metabolism.

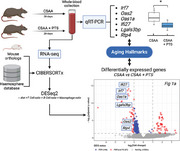

## 1 Introduction

Polyphenols from a group of phytoestrogens with a stilbene‐derived structure, such as resveratrol (RSV) and pterostilbene (PTS), among others, can exert pro‐health benefits in a variety of different disease models, including metabolic syndrome, type 2 diabetes, cardiovascular disease, and cancer.^[^ ^1^ ^]^ Their estrogen‐like properties have attracted special attention in hormone‐dependent cancers of breast and prostate origins. A myriad of beneficial health outcomes in response to phytoestrogens may arise through their antioxidant, anti‐inflammatory, and anti‐cancer effects, which were shown to be, at least partially, mediated by epigenetic mechanisms.^[^ ^1^ ^]^ Epigenetics refers to changes in gene expression without changes in the underlying DNA sequence^[^ ^2^ ^]^ and may be mechanistically responsible for phenotypic outcomes of phytoestrogens.^[^ ^3^, ^4^ ^]^ For instance, studies from our group showed that PTS attenuates breast cancer by epigenetic silencing of genes with oncogenic functions and works through regulating the interaction of epigenetic enzymes with specific DNA loci.^[^ ^4^ ^]^ In addition, recent preclinical studies have found that RSV and PTS may also confer increased longevity and lifespan, and slow down the aging of various model organisms.^[^ ^5^, ^6^ ^]^ Our previous work also showed that phytoestrogens are able to modify epigenetic patterns in healthy mammary epithelial cells, revealing for the first time a set of genes that respond to phytoestrogens in a healthy state.^[^ ^7^ ^]^ These genes were functionally linked to metabolism, immune functions, and signal transduction pathways.

Within the past decade, hematopoietic decline has emerged as one of the principal drivers of aging.^[^ ^8^, ^9^, ^10^ ^]^ Multiple studies discovered that blood and plasma transfer from young mice to old mice can rejuvenate multiple organs including the brain, liver, muscle, and heart.^[^ ^11^ ^]^ Recent findings demonstrate that the blood and tissues of rejuvenated mice have transcriptomic and epigenomic signatures of an intermediate phenotype between old and young animals.^[^ ^12^ ^]^ It is now known that hematopoietic function declines with age, which leads to the deterioration of immune and metabolic functioning. Hence, blood may reflect systemic effects of aging as demonstrated in transcriptomics, proteomics, and metabolomics studies.^[^ ^8^, ^9^, ^10^ ^]^ Transcriptomic profiling of human longevity and biological age in blood samples has identified differentially expressed genes that are involved in the regulation of the immune system, inflammatory processes, signal transduction, and metabolic pathways.^[^ ^13^, ^14^, ^15^, ^16^ ^]^ Importantly, phytoestrogens, including RSV, PTS, and quercetin, have been shown to exert effects on gene expression profiles in blood of patients with obesity/diabetes and coronary heart disease, affecting anti‐inflammatory pathways^[^ ^17^, ^18^ ^]^ and markers of oxidative stress.^[^ ^19^ ^]^ However, it remains unknown whether phytoestrogens impact the landscape of gene expression in blood in a healthy state and what processes may be affected.

In the present study, therefore, we conducted RNA sequencing to determine transcriptome‐wide changes in gene expression in whole blood of healthy rats to assess the role of phytoestrogen consumption on aging‐associated genes and propose that supplementation of the diet with phytoestrogens may be able to induce changes in gene expression that ultimately confer pro‐longevity benefits. Interestingly, extensive studies on RSV and PTS demonstrated that PTS, as compared to RSV, is characterized by greater bioavailability, hepatic stability, and bioactivity, which positions PTS as a superior candidate for further investigations.^[^ ^20^ ^]^ However, whether PTS exerts pro‐longevity benefits comparable to RSV has yet to be confirmed. Hence, we assessed both PTS and RSV at the same molar concentrations in the present study. We also included chlorogenic acid (CGA), a non‐estrogenic polyphenol found in coffee beans, with potent health effects that could surpass those of RSV.^[^ ^21^ ^]^ This allows us to compare the studied effects between compound with different chemical structure.

## 2 Results

### 2.1 Ortholog Deconvolution Identified Transcriptome Changes in Rats on pterostilbene (PTS)‐Supplemented Diet

Exploratory data analysis on the whole‐blood samples (mixture data) after filtering for lowly expressed genes confirmed homogeneous count distributions for most of the samples (Figure S1A, Supporting Information). One sample from the RSV diet was identified as an outlier due to a high number of genes having unusually low expression values (Figure S1A, Supporting Information). This sample was removed from downstream analyses. Principal component analysis (PCA) was employed on the complete expression profiles of all samples to determine the main sources of gene expression variability in the mixture data. PC2 showed a separation between control CSAA diet and PTS‐supplemented CSAA diet, while other supplemented groups, RSV and CGA, were mostly clustered with the reference CSAA group (Figure S1B, Supporting Information). PCA performed exclusively on samples from CSAA diet and PTS supplemented diet still showed that the expression variability associated with dietary differences accounts for 30.05% of the observed variability (Figure S1C, Supporting Information).

Differential expression analysis of the mixture data (no deconvolution) identified two DEGs when comparing CSAA diet and PTS‐supplemented diet (Figure S2, Supporting Information), *Hba‐a1* and *Ifi27*, as depicted in red dots distributed above the threshold line of absolute fold change above 2 with adjusted *p‐*value ≤ 0.05 (Figure S2B, Supporting Information). In addition, nine genes showed smaller changes in magnitude however statistically significant, namely *Csnk2b*, *Lgals3* *bp*, *Atp2a3*, *Sf3a2*, *Srpra*, *Adar*, *Golga2*, *Rtp4*, and *Klhl2*. There were no significant differences for the other dietary comparisons: CGA versus CSAA or RSV versus CSAA. As suggested by the PCA and sample–sample correlation heatmaps, this low number of differentially expressed genes could result from subtle changes in the transcriptome of whole blood DNA of healthy rats. Since whole blood is a heterogenous mixture of multiple cell types, we decided to explore if the PTS‐related dietary effects on healthy rats are confounded with the multiple cell type proportions in whole blood. For this reason, we applied a cell‐type deconvolution pipeline to account for different proportions in four major cell types: B‐cells, T‐cells, macrophages, and neutrophils. The expectation is that cell‐type deconvolution will account for expression changes associated with variability in whole‐blood cell type proportions.

**Figure 1:**
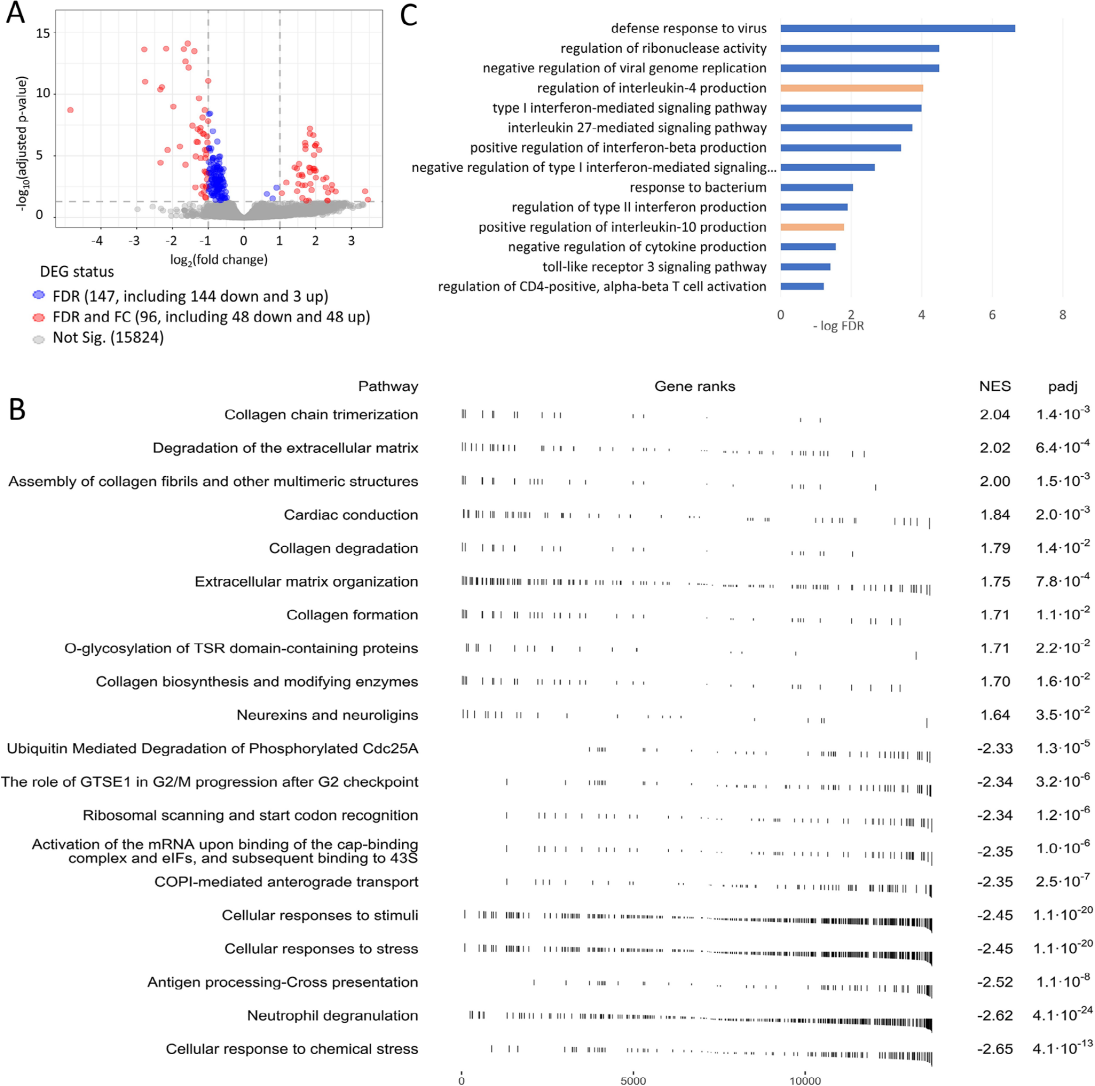
Ortholog cell deconvolution results. A) Volcano plot for deconvoluted differential expression results. Red dots show differentially expressed genes (DEGs), defined by an absolute fold change greater than two, and an adjusted *p*‐value below the significance threshold of ≤0.05 Genes shown in blue met the significance threshold but their change in expression was 2‐fold or below. In grey, genes that did not meet the significance threshold. We can observe an overall trend for reduced expression in the PTS supplemented diet. B) Gene set enrichment analysis showing the top enriched Reactome pathways in the results from the differential expression analysis. C) Significant Gene Ontology (GO) terms associated with differentially expressed genes in blood of rats on a diet supplemented with PTS. Only “Regulation of IL‐4 and IL‐10 production” terms are associated with upregulated genes (pink color); all other terms are liked to downregulated genes (blue color). GO analysis was performed using PANTHER database.^[^ ^23^ ^]^

A major limitation for cell deconvolution of rat whole‐blood is the lack of reference expression profiles for blood cell types in rat. For this reason, we identified a set of 9096 high‐confidence orthologs with available expression for sorted blood cell types in the Haemosphere database for B‐cells, T‐cells, macrophages, and neutrophils.^[^ ^22^ ^]^ The mouse expression profiles for the 9096 high‐confidence orthologs across B‐cells, T‐cells, macrophages, and neutrophils were used in CIBERSORTx to identify which genes best characterize expression differences between those cell types. This step produces a matrix with the relative importance from each gene to identify each cell type. This matrix is referred by CIBERSORTx as “signature matrix.” The signature matrix was then used in conjunction with the whole‐blood rat mixture dataset to determine the cell proportions using CIBERSORTx. Analysis of the estimated cell proportions identified T‐cells to be a major component in our samples, averaging 55% sample composition, and neutrophils were the least abundant, averaging 5% sample composition (Figure S3A, Supporting Information). We used the estimated cell scores as covariates for differential expression analysis, identifying 94 additional DEGs in addition to two DEGs found prior to deconvolution (**Figure** **1**A). Red dots in Figure 1A correspond to the 96 DEGs with adjusted *p* ≤ 0.05 and an absolute fold change of greater than 2. We verified that DEGs shared with the signature matrix had low weight values for identifying the different cell proportions (Figure S3B, Supporting Information). Genes were ranked based on the weight values, i.e., the first ranks were assigned the most importance during cell deconvolution by CIBERSORTx. Twelve DEGs overlapped with the signature matrix, spanning from rank 107 to 1156, based on their relevance to identify the reference blood types (as shown by the red lines in Figure S3B, Supporting Information). Given the exponential decay on weights, the first 50 ranks had the highest contribution during deconvolution, suggesting that genes with observed expression changes upon exposure to a PTS‐supplemented diet are not directly influenced by the deconvolution pipeline. A gene set enrichment analysis using the Reactome pathways identified upregulated pathways associated with intercellular interactions, and remodeling of the extracellular matrix, while downregulated pathways were mostly associated with cellular stress response (Figure 1B). Importantly, Gene Ontology (GO) analysis of top differentially expressed genes, i.e., with fold change >2, revealed anti‐inflammatory IL‐4 and IL‐10 production to be associated with upregulated genes (Figure 1C, pink color), while pro‐inflammatory interferon‐mediated signaling pathways are linked to genes downregulated in response to PTS (Figure 1C, blue color).

### 2.2 The Landscape of Changes in Blood mRNA Levels in Response to Pterostilbene (PTS)

**Figure 2:**
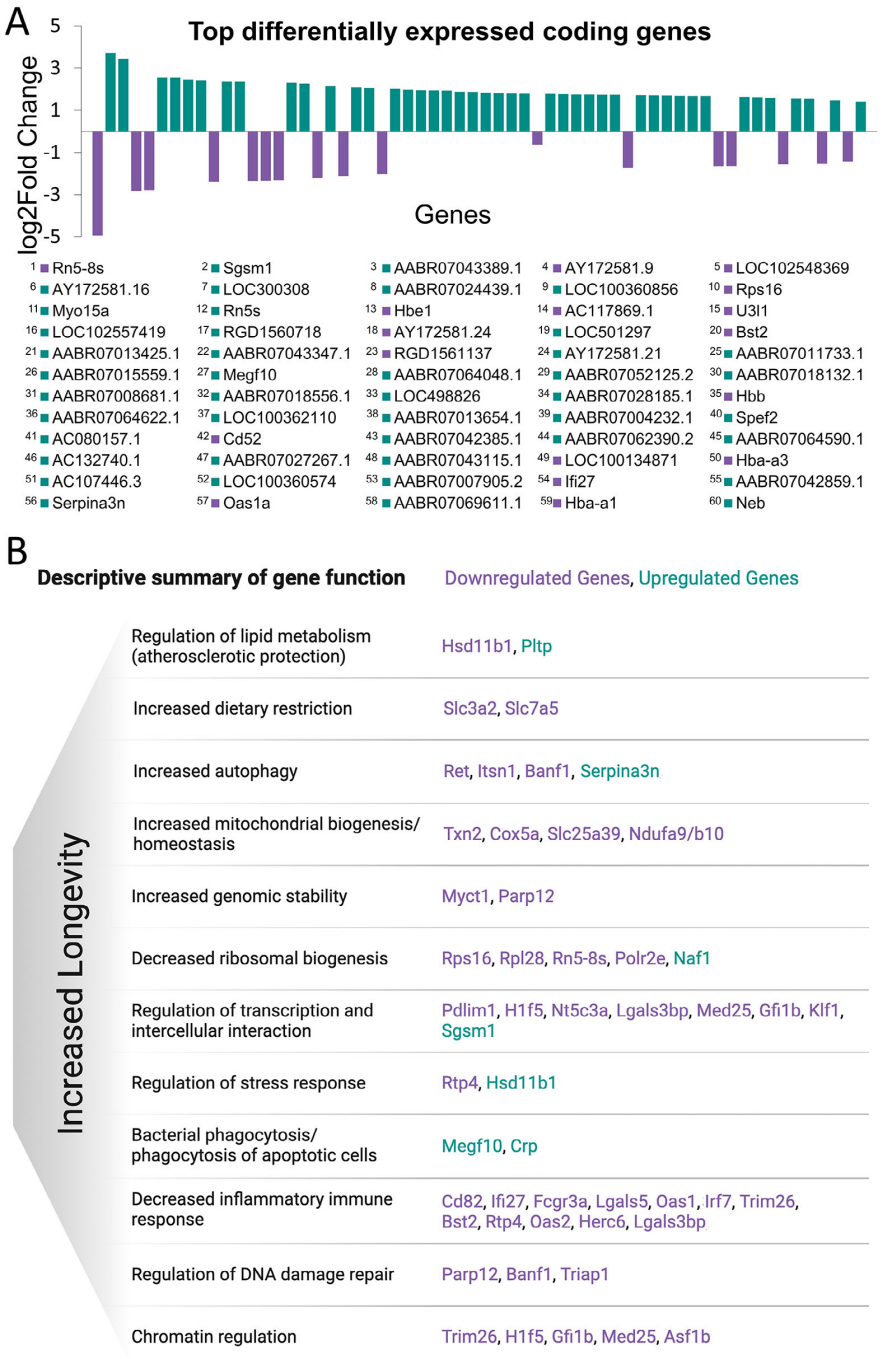
Transcriptomic changes in blood of rats exposed to pterostilbene (PTS)‐supplemented diet. A) Fold change values, expressed as log2(Fold Change), for the top 60 differentially expressed genes (DEGs) in PTS‐supplemented rats as compared to CSAA rats. Violet color corresponds to downregulated genes (negative value) whereas upregulated genes are marked in turquoise (positive value). Genes are numbered from the highest to the lowest (|log2(FC)| value. B) A descriptive summary of functions associated with differentially expressed genes (PTS‐target genes, fold change ≥1.5), as assessed by a literature search in PubMed. Functions associated with at least two genes are depicted. Downregulated genes are in violet and upregulated genes are in turquoise. The picture was created in BioRender.

Exposure of rats to diet supplemented with PTS resulted in statistically significant changes of expression levels of 243 genes in collected blood, after the deconvolution pipeline (Table S2, Supporting Information). The majority of changes, 192/243, demonstrated decrease in expression and the magnitude of the difference was greater than 2 in 48 out of 192 genes (Figure 1A). Among 144 downregulated genes with an absolute fold change of 2 and lower than 2 (FDR status, Figure 1A), only 14 fell below 1.5‐fold change. The remaining 51 genes out of 243 DEGs were upregulated and the increase in expression was greater than 2 in 48 of them (Figure 1A). Although overall more DEGs were downregulated, the majority of high‐magnitude changes were among upregulated genes, as shown in **Figure** **2**A depicting 60 top changes.

Interestingly, the top downregulated genes, *Rn5‐8s* and *Rps16*, encode components of the ribosome. In eukaryotes, the ribosome is the translation machinery and is composed of two subunits, four rRNAs, and multiple ribosomal proteins.^[^ ^24^ ^]^ The 5.8S rRNA is part of the large subunit along with 28S and 5S rRNAs. Rps16, in turn, is a ribosomal protein that is a component of the small subunit of the ribosome that contain 18S rRNA.^[^ ^24^ ^]^ A profound amount of energy is used for biogenesis of ribosomes to translate RNA into proteins. Reduction in energy‐consuming processes such as ribosomal biogenesis has been shown as a fundamental mechanism for extending lifespan.^[^ ^24^ ^]^

On the other hand, 5S rRNA (Rn5s) is among upregulated genes with 5‐fold increase in expression. Although it seems contradictory, it is important to note that 5.8S and 5S rRNAs are transcribed from distinct DNA loci and the function of 5S rRNA is unique.^[^ ^25^ ^]^ It forms a pre‐ribosomal complex with the ribosomal protein L5 prior to joining the ribosome. The interaction with L5 is required for the transport of 5S rRNA into the nucleolus where the ribosome will be assembled.^[^ ^25^ ^]^ Of importance, 5S rRNA plays a crucial role in the formation of a physical connection between the different regions of the ribosome. It may sense the amounts of other rRNAs and ribosomal proteins.^[^ ^25^ ^]^ Thus, upregulation of 5S rRNA could be a compensatory response to lower levels of 5.8S rRNA and ribosomal proteins.

The highest increase in mRNA levels was detected for *Sgsm1*, which is a modulator of small G‐protein signaling, predicted to be involved in regulation of transcription by RNA polymerase II.^[^ ^26^ ^]^ Importantly, reduced SGSM1 expression was linked to metastasis in nasopharyngeal carcinoma^[^ ^27^ ^]^ and was correlated with poor overall survival in gliomas, indicating SGSM1 anti‐oncogenic functions.^[^ ^26^ ^]^

### 2.3 Differentially Expressed Genes in Response to Pterostilbene (PTS) Are Associated with Functions that Counteract Hallmarks of Aging

Using existing evidence in literature for every gene individually from differentially expressed genes (fold change ≥ 1.5), we have performed a detailed functional analysis and depicted functions associated with at least two genes in Figure 2B. Recent findings emphasize 10 hallmarks of aging.^[^ ^28^ ^]^ Seven of those hallmarks are counteracted by functions of genes that are differentially expressed in response to PTS supplementation in our study. Those include: genomic instability (increase in genomic stability by PTS‐mediated changes), epigenetic alterations (regulation of chromatin by PTS), compromised autophagy (increased autophagy by PTS‐mediated changes), mitochondrial dysfunction (increased mitochondrial biogenesis by PTS), deregulated nutrient sensing (increased dietary restriction by PTS), altered intercellular interaction (regulation of cell–cell and cell–matrix interaction by PTS), and loss of proteostasis (phagocytosis and decreased ribosomal biogenesis by PTS) (Figure 2B). Hence, PTS‐target genes are enriched with functions that are tightly connected to slowing down aging and potentially increasing lifespan.

A decrease in the inflammatory immune response is a category that encompasses the highest number of differentially expressed genes, including genes from the interferon signaling pathway. One of them is *Ifi27*, the interferon alpha inducible protein 27, that is downregulated upon PTS supplementation. Another downregulated gene with functions related to Ifi27 is *Irf7*, the interferon regulatory transcription factor. Both *Ifi27* and *Irf7* play a pro‐inflammatory role and their upregulation is associated with driving inflammatory conditions.^[^ ^29^, ^30^ ^]^ Of relevance, IFI27 has been found to be significantly overexpressed in individuals between ages 90 and 99 as compared to middle‐age controls, raising a possibility that IFI27 may be implicated in regulation of longevity and healthy aging.^[^ ^13^ ^]^ IRF7, in turn, was upregulated in aging adipose‐derived stromal cells which was driving aging‐associated metabolic dysfunction.^[^ ^31^ ^]^ Downregulation of *Lgals3* *bp*, which is implicated in the immune response, may also create a functional bridge to lengthen lifespan. LGALS3BP protein level was found to be lower in middle‐aged people compared to those 90–99 years of age^[^ ^13^ ^]^ and in men who lived longer as compared to those who died earlier, within 12 years of follow‐up.^[^ ^32^ ^]^

In the group of PTS‐downregulated genes that functionally drive inflammatory response, we detected two interesting candidates, *Oas2* and *Oas1a*. *OAS* genes inhibit virus replication through the activation of RNA cleavage pathway and have been demonstrated to enhance secretion of pro‐inflammatory cytokines in infectious disease^[^ ^33^ ^]^ and contribute to inflammation in psoriasis.^[^ ^34^ ^]^ Importantly, low doses of anti‐viral chloroquine decreased levels of *Oas1a* and *Oas2* in aged rats which was accompanied by extended lifespan.^[^ ^35^ ^]^ *Oas2* was also among the genes overexpressed in the liver and kidney of older rats compared to younger rats.^[^ ^36^ ^]^

Healthy longevity has also been shown to be promoted by dietary restriction and limited essential amino acid intake.^[^ ^37^, ^38^ ^]^ PTS‐mediated downregulation of transporters such as *Slc7a5* and *Slc3a2* that mediate uptake of amino acids, including essential amino acids, may facilitate dietary restriction and thus increase longevity.^[^ ^39^ ^]^

Decreased levels of amino acids have been shown to be sensed by the integrated stress response (ISR) pathway that activates pro‐longevity genes (e.g., FGF21).^[^ ^37^ ^]^ However, the active ISR pathway has also been linked to cognitive decline in aging.^[^ ^40^ ^]^ Inhibition of ISR improved cognitive functions and was accompanied by decreased expression of *Rtp4* in hippocampal samples of aged mice.^[^ ^40^ ^]^ Interestingly, downregulation of *Rtp4* is mimicked by PTS supplementation in our study (Figure 2B). Regulation of stress response signaling molecules is also executed by another gene in this category, namely *Hsd11b1*. This gene encodes for hydroxysteroid 11‐beta dehydrogenase which catalyzes the reversible conversion of the inactive metabolite cortisone to the stress hormone cortisol, the main glucocorticoid.^[^ ^41^ ^]^ Although HSD11B1 impairs anti‐tumor immune response due to rising levels of glucocorticoids^[^ ^42^ ^]^ and increased expression of HSD11B1 in aged skin leads to impaired skin barrier function,^[^ ^43^ ^]^ the effects of HSD11B1 are complex and include anti‐inflammatory activity in skin cells, as well.^[^ ^44^ ^]^ Considering latest evidence for pro‐longevity effects of *Hsd11b1* inhibition in rodents and in pilot trials in healthy elderly people,^[^ ^41^ ^]^ PTS‐mediated increase of *Hsd11b1* expression in rat blood may have more diverse effects, possibly focused on homeostasis between anti‐ and pro‐inflammatory signals.

Apart from regulating the stress response, *Hsd11b1* has also been implicated in regulation of lipid metabolism. Of note, improved lipid metabolism has been positively linked to familial longevity.^[^ ^45^ ^]^ Deficiency in *Hsd11b1* brings about cardioprotective lipid profile in some studies whereas other studies show no modulation of atherosclerosis susceptibility upon HSD11B1 inhibition.^[^ ^46^ ^]^ It is possible that PTS increases *Hsd11b1* expression in our study primarily to suppress inflammation by acute release of glucocorticoids. Within the group of genes linked to lipid metabolism, we also identified *Pltp*, a phospholipid transfer protein that is reduced by PTS. *Pltp* regulates lipid metabolism by increasing transfer of phospholipids and cholesterol from lipoproteins to HDL and thus increasing the size of HDL.^[^ ^47^ ^]^ *PLTP* was one of the genes significantly upregulated in blood specimens and linked to a favorable lipid profile in centenarians.^[^ ^45^ ^]^ Thus, the role of *Pltp* downregulation by PTS in blood of rats in our study is not clear and may be linked to homeostasis in inflammatory signaling pathways.

Chromatin regulation is another interesting category represented by *Asf1b* and *Gfi1b*, among others, that were downregulated in response to PTS (Figure 2B). Elevated histone chaperone ASF1B brings about oncogenic effects in cancer.^[^ ^48^, ^49^ ^]^ Its involvement in longevity is supported by the evidence demonstrating that reduced expression of another member of the ASF1 family, *ASF1A*, correlates with familial longevity, which may result from alterations in histone modifications.^[^ ^13^ ^]^ Indeed, changes in chromatin state have been shown to be the key event of mammalian aging.^[^ ^50^ ^]^ GFI1B is a DNA binding transcriptional regulator implicated in hematopoietic differentiation.^[^ ^51^ ^]^ Along with lysine‐specific demethylase, KDM1A, GFI1B recruits histone deacetylase to suppress transcription of numerous genes controlling hematopoiesis.^[^ ^52^, ^53^ ^]^ As for ASF1B, abnormal activation of GFI1B contributes to blood malignancies.^[^ ^54^ ^]^

### 2.4 Validation of Changes in Expression of Candidate Genes in Response to Pterostilbene (PTS), Using qRT‐PCR

**Figure 3:**
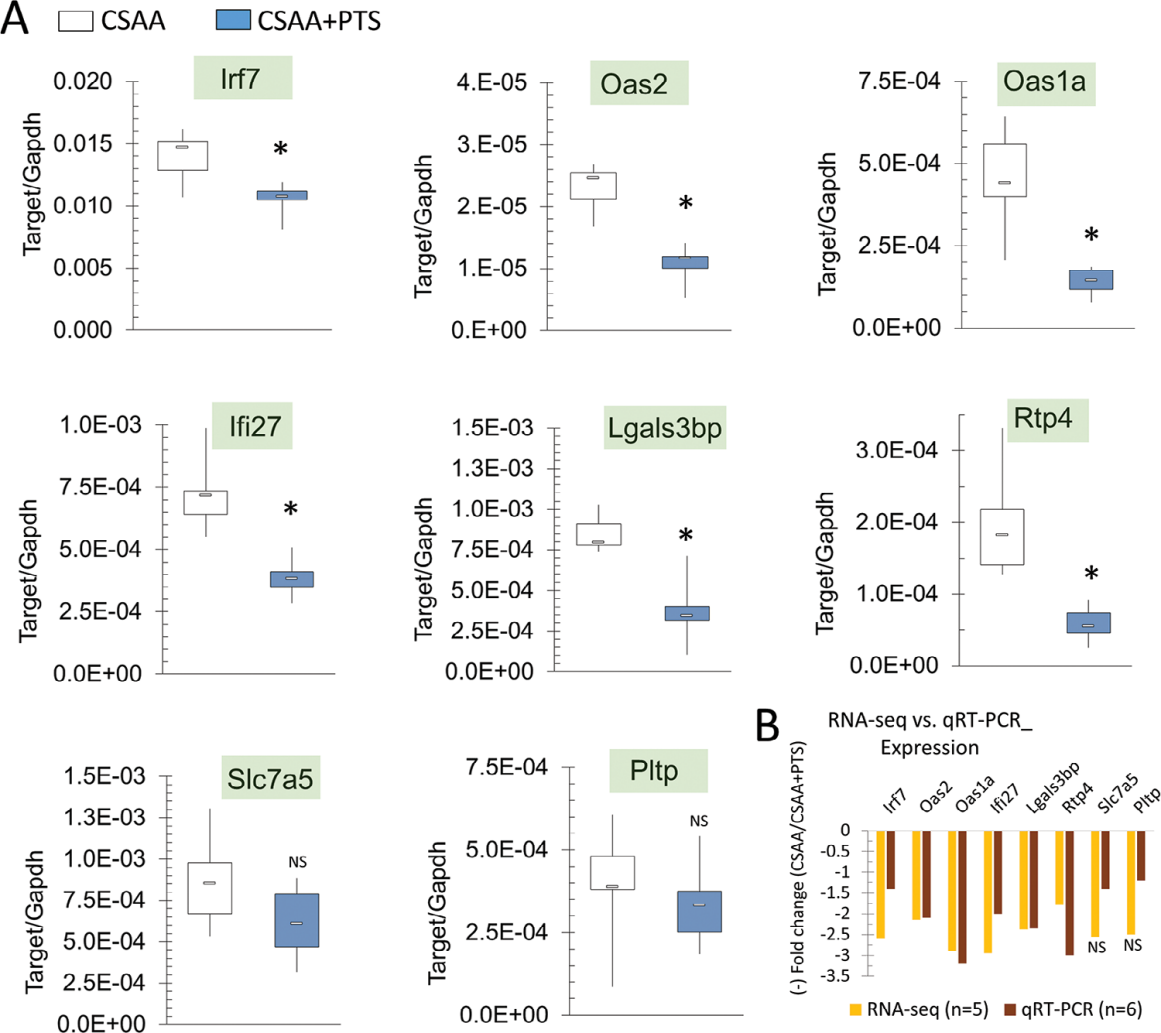
Quantitative validation of transcriptomic changes in blood of rats exposed to pterostilbene (PTS)‐supplemented diet for the selected PTS‐target genes. A) Downregulation of *Irf7*, *Oas2*, *Oas1a*, *If*i27, *Lgals3* *bp*, *Rtp4*, *Slc7a5*, and *Pltp* in PTS‐supplemented group of rats (CSAA+PTS) versus CSAA control group, as measured by qRT‐PCR. All results expressed in a boxplot as min, IQR, and max of target/Gapdh; *n* = 6 per group; *p < 0.05. Statistical analysis of qRT‐PCR was performed using Mann–Whitney *U* test. B) Fold‐change in expression values (CSAA+PTS/CSAA) for the selected PTS‐target genes as measured by RNA‐seq (*n* = 5) or qRT‐PCR (*n* = 6). Fold change is shown as a negative value to reflect downregulation of the selected genes in CSAA+PTS versus CSAA.

Using qRT‐PCR, we have validated changes in gene expression for several genes from key functional categories such as inflammatory immune response (*Irf7*, *Oas2*, *Oas1a*, *Ifi27*), regulation of intercellular interaction (*Lgals3* *bp*), stress response (*Rtp4*), dietary restriction (*Slc7a5*), and lipid metabolism (*Pltp*) (**Figure** **3**A). Of importance, the selected genes were linked to regulation of longevity and aging, as described in the paragraph above.

There was an association between changes detected in RNA‐seq and qRT‐PCR considering the direction and magnitude of a change (Figure 3A,B). Although downregulation of *Slc7a5* and *Pltp* was statistically non‐significant, the direction of the change was maintained. For the remaining genes, both techniques detected significant changes in the same direction (Figure 3B). The concordance between qRT‐PCR results and the candidate DEGs identified by the ortholog‐deconvolution pipeline further supports using high‐confidence orthologs to leverage information from mouse datasets.

### 2.5 Assessing Expression of the Selected Pterostilbene (PTS)‐Target Genes in Human Tissues

Functional analyses of genes that were differentially expressed in blood of rats exposed to PTS (i.e., PTS‐target genes) suggest a possible impact on longevity and healthy aging. Therefore, we have hypothesized that PTS‐target genes may be differentially expressed in humans depending on their biological age. Using publicly available data in the Gene Expression Omnibus (GEO), we identified GSE67220 dataset of RNA‐seq in whole blood of young (46 ± 3 years, *n* = 11) versus aged (68 ± 4 years, *n* = 9) individuals. The blood expression profile of the selected PTS‐target genes that were validated by qRT‐PCR (Figure 3) did not show any significant differences in this dataset (data not shown). The lack of age‐dependent changes in gene expression in the blood may result from the low number of research participants and/or the fact that the chronological age of patients does not always reflect their biological age.^[^ ^8^ ^]^ Moreover, the discordance of cohorts in terms of biological sex, genetic ancestry, ethnicity, and other demographic characteristics may mask the changes in gene expression. Cell type composition changes in whole blood samples may come into play as well.

**Figure 4:**
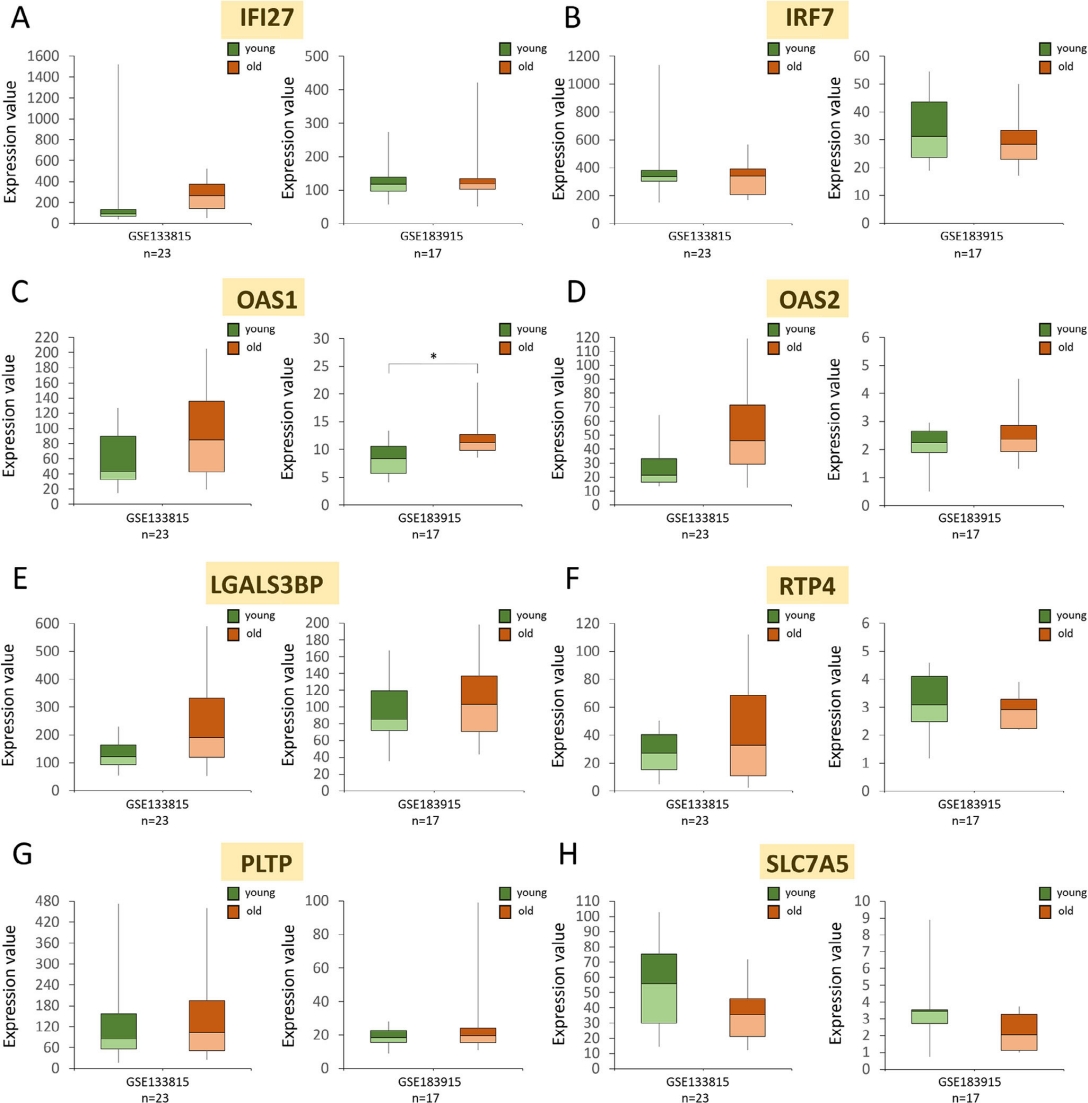
Expression of selected PTS‐target genes in human liver tissue from healthy individuals in different age groups. A‐H) Expression of the selected PTS‐target genes in young (<49 years of age) versus older (>74 years of age) adults, based on the Affymetrix array (GSE133815) and RNA sequencing (GSE183915) from publicly available datasets in the Gene Expression Omnibus (GEO) database. Results expressed as min, IQR, and max of the array intensity or RNA‐seq value (expression value); *p < 0.05. Patient characteristics corresponding to the data are depicted in Table S3, Supporting Information.

**Figure 5:**
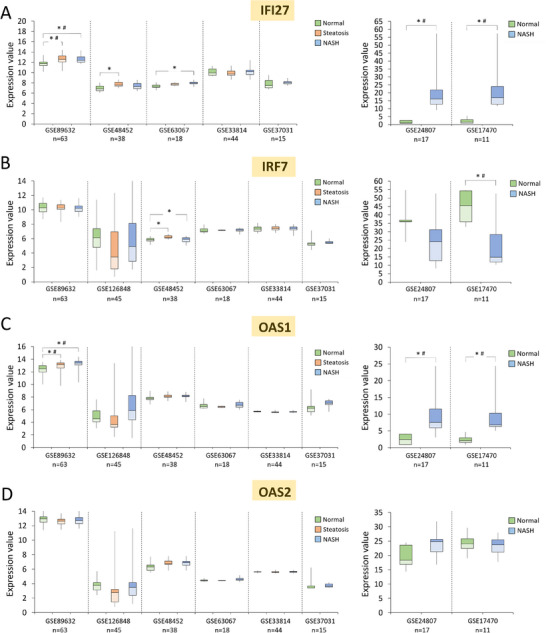
Expression of IFI27, IRF7, OAS1, and OAS2 in human liver tissues from healthy individuals and patients with steatosis or non‐alcoholic steatohepatitis (NASH). A–D) Expression of IFI27, IRF7, OAS1, and OAS2 in the liver of healthy individuals (Normal) and patients with steatosis or NASH, based on the Affymetrix, Illumina, or CodeLink Human Whole Genome array data from publicly available datasets in the Gene Expression Omnibus (GEO) database. Results expressed as min, IQR, and max of the array intensity (expression value); *p < 0.05; ^#^adjusted *p* < 0.05. Patient characteristics corresponding to the data are depicted in Table S4, Supporting Information.

**Figure 6:**
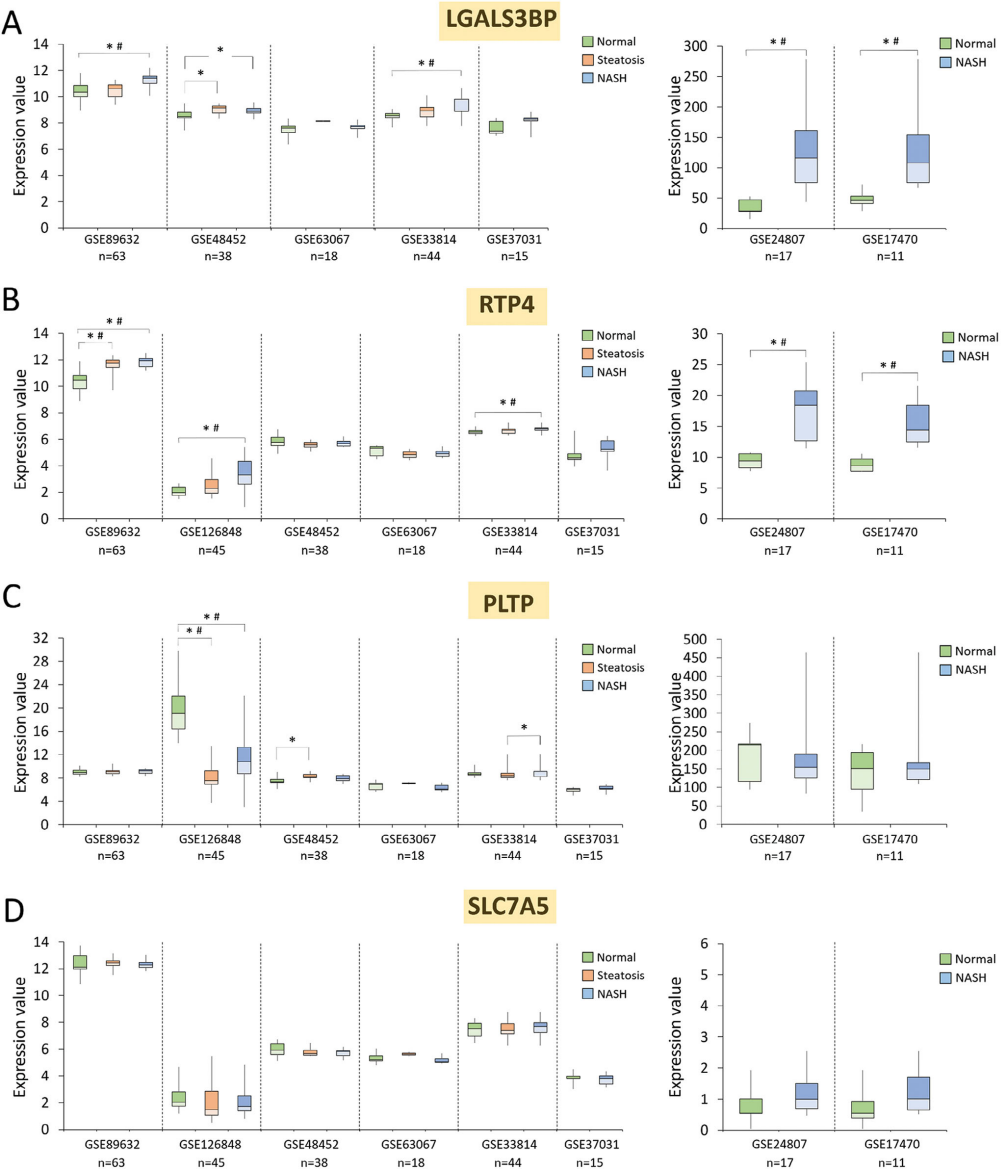
Expression of LGALS3BP, RTP4, PLTP, and SLC7A5 in human liver tissues from healthy individuals and patients with steatosis or non‐alcoholic steatohepatitis (NASH). A–D) Expression of LGALS3BP, RTP4, PLTP, and SLC7A5 in the liver of healthy individuals (Normal) and patients with steatosis or NASH, based on the Affymetrix, Illumina or CodeLink Human Whole Genome array data from publicly available datasets in the Gene Expression Omnibus (GEO) database. Results expressed as min, IQR, and max of the array intensity (expression value); *p < 0.05; ^#^adjusted *p* < 0.05. Patient characteristics corresponding to the data are depicted in Table S4, Supporting Information.

Aging is associated with impaired functions of the liver which is a central organ in regulation of cellular metabolism.^[^ ^55^ ^]^ Disturbed metabolism, including dysfunctional lipid metabolism and the inability of the liver to transport lipids to peripheral tissues, has been mechanistically connected to accelerated aging.^[^ ^56^ ^]^ Hence, we have analyzed expression of the selected PTS‐target genes in the liver tissue of young versus aged individuals (**Figure** **4**), as well as in individuals with steatosis or non‐alcoholic steatohepatitis (NASH) (**Figures** **5** and **6**). Of note, steatosis is fat accumulation in the liver, which compromises its functioning but without signs of liver inflammation or damage. The latter two features are characteristic of NASH that is the most severe type of non‐alcoholic fatty liver disease (NAFLD).^[^ ^57^ ^]^

The selected PTS‐target genes are downregulated upon PTS supplementation which would imply their upregulation in aging liver and in a disease state such as fatty liver disease. Indeed, we detected increases in expression of some of the selected PTS‐target genes in livers from older adults (>74 years of age) as compared to young adults (<49 years of age) (Figure 4). In GSE133815 dataset, mRNA increases were detected for six out of eight genes, for instance 3‐fold increase for *IFI27* (Figure 4A) and 2‐fold increase for *OAS1* and *OAS2* (Figure 4C,D) in older versus young individuals, however no changes were statistically significant (*p* > 0.05) (Figure 4). In another dataset of RNA‐seq of liver tissue, GSE183915, statistically significant upregulation was observed only for *OAS1* with 35% increase in the median expression in older versus young individuals (*p* < 0.05; adj. *p* > 0.05) (Figure 4C).

Interestingly, PTS‐target genes also showed a pattern of increased mRNA levels in liver tissue of individuals diagnosed with different stages of fatty liver disease (Figure 5). In GSE89632 dataset, with the highest number of participants (n = 63), *IFI27* was significantly upregulated by 7% and 5.4% in steatosis and NASH, respectively, as compared with healthy individuals (normal) (adj. *p* < 0.05) (Figure 5A, left panel). Among the remaining six datasets, four of them showed *IFI27* upregulation in NASH as well, with the highest and statistically significant increase after the adjustment for multiple comparisons in GSE24807 (16.5‐fold increase) and GSE17470 (11‐fold increase) (Figure 5A, right panel). *OAS1* is consistently upregulated in NASH compared with healthy individuals and the change reaches statistical significance in three out of eight datasets (adj. *p* < 0.05) (Figure 5C). In GSE89632 dataset with the highest number of participants, *OAS1* expression is significantly upregulated also in steatosis versus healthy (Figure 5C). As observed for *IFI27*, the highest 3‐fold increase in *OAS1* expression in NASH is reported in GSE24807 and GSE17470 datasets (Figure 5C, right panel). Expression of *IRF7*, which is another candidate implicated in driving inflammatory response, was significantly increased by 2.1% in one dataset, GSE48452 (*p* < 0.05; adj. *p* > 0.05) (Figure 5B). Another dataset, GSE17470, unexpectedly showed *IRF7* mRNA level to be significantly decreased (adj. *p* < 0.05) (Figure 5B, right panel). Of note, GSE17470 had the smallest number of participants (*n* = 11) among the eight datasets.

Among genes engaged in regulation of intercellular interaction (*Lgals3* *bp*), stress response (*Rtp4*), lipid metabolism (*Pltp*), and dietary restriction (*Slc7a5*) (Figure 6), *LGALS3BP* expression rises in NASH versus normal (healthy individuals), with adj. *p* < 0.05 in four out of eight datasets (Figure 6A). Similarly, *RTP4* is significantly upregulated in NASH in five datasets as well as in steatosis in one of the GEO entries (adj. *p* < 0.05) (Figure 6B). The magnitude of changes is the largest in GSE24807 and GSE17470. *LGALS3BP* and *RTP4* are upregulated by 2.3–4‐fold and 1.7–2‐fold, respectively, in these sets (Figure 6A,B, right panels). Interestingly, an increase in expression of *PLTP* in steatosis or NASH was detected in two datasets, GSE48452 and GSE33814 (*p* < 0.05; adj. *p* > 0.05), whereas GSE126848 showed statistically significant downregulation of *PLTP* in both steatosis and NASH (adj. *p* < 0.05) (Figure 6C).

## 3 Discussion

Differentially expressed genes in the blood samples of rats on a diet supplemented with PTS, as compared with a control CSAA diet, are enriched with multiple pathways (Figure 1B,C) and processes (Figure 2B) that counteract aging.^[^ ^28^ ^]^ Among hallmarks of aging, impaired autophagy, mitochondrial dysfunction, and loss of proteostasis are major processes that emerge in the immune system and drive age‐related decline of the immune functions, leading to low‐grade chronic inflammation, so called inflammaging, in the blood and tissues.^[^ ^58^ ^]^ In turn, inflammaging results in disturbances in metabolism, including immune cell metabolism, which further exacerbates inflammatory responses controlled by the immune system. Importantly, most changes induced by PTS in gene expression in the blood in our study are linked to a decreased inflammatory response, particularly of the innate immune system and interferon pathway (Figure 2B). Indeed, many interferon‐related genes upregulated during viral infection in rats were found to be downregulated by PTS in this study (e.g., *Oas1a*, *Oas2*, *Irf7*, *Ifi27*, *Lgals3* *bp*). These changes may indicate a shift in macrophage polarization from pro‐inflammatory M1 to anti‐inflammatory M2 types.^[^ ^59^ ^]^ Indeed, previous studies showed that PTS can depolarize M1 macrophages or microglia and promote their transition to anti‐inflammatory M2 macrophage phenotypes.^[^ ^60^ ^]^ Since resident macrophages of certain tissues appear to preferentially polarize to M1 types with aging.^[^ ^61^, ^62^ ^]^ PTS‐induced shift to M2 macrophages would imply prevention of age‐associated inflammatory immune phenotypes. Importantly, our gene ontology analysis (Figure 1C) confirms a decrease in M1‐activating type II interferon (IFNγ) and an increase in M2‐activating IL‐4, which further suggests the change to the anti‐inflammatory state upon PTS supplementation.^[^ ^59^ ^]^ Apart from IL‐4, another anti‐inflammatory cytokine, IL‐10, is associated with PTS‐upregulated genes, which could contribute to maintaining the balance of the immune response. Of note, it has been proposed that over the course of a lifetime, reduction in inflammation may prolong longevity.^[^ ^63^ ^]^ Interestingly, reducing constitutive levels of the antiviral RNA sensor oligoadenylate synthetase, *Oas1*, one of the downregulated genes in this study, can promote longevity in mice.^[^ ^64^ ^]^

PTS may also further promote anti‐inflammatory immune phenotypes and cellular homeostasis by activating autophagy and related processes such as endoplasmic reticulum delivery into autophagosomes (so called ERphagy), as indicated in Figure 2B. Indeed, macrophages of aged mice show decreased autophagic ability and increased anti‐autophagic mTOR signaling, resulting in a greater propensity to polarize to pro‐inflammatory M1 macrophages upon inflammatory stimuli.^[^ ^65^ ^]^ In our study, PTS supplementation strongly reduced expression of *Rn5‐8s* (Figure 2A), a highly abundant ribosome subunit transcribed by RNA Pol III, whose levels are thought to reflect cell stress and mTOR activity.^[^ ^66^ ^]^ Additionally, ribosome biogenesis contributes to immune cell activation and innate immune anti‐viral functions.^[^ ^67^ ^]^ Thus, PTS may halt immune cell activation through immunometabolism, i.e., changes in cellular metabolism that affect immune cell functions.^[^ ^68^ ^]^ A further autophagy‐promoting signal mediated by PTS in our study results from downregulation of *Slc7a5* (Figure 2A) that regulates mTOR activity through the increased uptake of essential amino acids; the knockdown of *Slc7a5* hinders amino acid uptake, potentially signaling dietary restriction^[^ ^39^ ^]^ prevents mTORC1 activation, and enhances autophagy.^[^ ^39^ ^]^ Importantly, previous studies showed that the PTS analog, RSV, mimics the effects of dietary restriction in mice and leads to decreased expression of pro‐inflammatory *Ifi27* in the liver and other organs, indicating suppression of inflammatory responses.^[^ ^69^ ^]^ PTS treatment also reduced the expression of *Itsn1* gene encoding for one of key inhibitors of autophagosome formation and trafficking (Figure 2B). Overall, by promoting autophagy, PTS gains the ability to maintain immune cells in a quiescent state to avoid spontaneous interferon production and excessive inflammation, to promote balanced proteostasis, and to prevent the accumulation of dysfunctional mitochondria and proteins, which can rescue a cell from increased ROS production, decreased respiratory functioning, and increased pro‐apoptotic signaling.^[^ ^70^ ^]^

As shown in Figure 1C, type 1 interferon‐mediated signaling pathway, that plays a role in adaptive immunity cell maturation and activation, is likely inhibited by PTS in our study. Findings by Shabani et al. echo this conclusion as they found that another phytoestrogen with stilbene rings, RSV, decreases M1 macrophage polarization and increases T_Reg_ cell population in a mouse model of obesity.^[^ ^71^ ^]^ Indeed, PTS has been previously shown to promote T_Reg_ differentiation in a dendritic cell‐dependent manner.^[^ ^72^ ^]^ The beneficial effects of phytoestrogens can therefore be clinically useful to treat conditions exhibiting overactive immune inflammation, such as irritable bowel disease (IBD) and multiple sclerosis (MS), which likely develops because of insufficient T_Reg_s. However, whether these compounds can act as general longevity prophylactics or only as anti‐inflammatories in cases of pre‐existing inflammation and immune dysregulation, remains to be seen. Surprisingly, certain “reverse interferon” signatures like those found here, which might be expected to be anti‐inflammatory, can contribute to the development of spondyloarthritis in rats.^[^ ^73^ ^]^ As such, long‐term consumption of these compounds may not necessarily be health‐promoting under all circumstances. Nevertheless, we also observed downregulation of *Rtp4* that has recently been involved in the inflammatory response.^[^ ^74^ ^]^ *Rtp4* is induced by type 1 interferon in infections and consequently interferes with interferon signaling pathways. *Rtp4*‐knockout mice have higher levels of type 1 interferon response specifically in the brain and better survival upon infection with malaria parasites.^[^ ^74^ ^]^ Thus, PTS‐mediated decrease in *Rtp4* expression may have a regulatory effect and contribute to homeostasis in inflammatory signaling pathways, considering dampened inflammatory response due to changes in other interferon‐related genes.

While the direct effects of PTS on the immune system can account for the majority of the observed effects on interferon signaling and macrophage polarization genes, evidence suggests that decreases in interferon‐related genes can stem from alterations in the microbiota following phytoestrogen antioxidant incorporation into the diet.^[^ ^71^ ^]^ Indeed, Stefan et al. noted reductions in type 1 interferon (IFN‐I) and *Irf7* levels, like in our study, following microbiome modifications in mice.^[^ ^75^ ^]^ *Irf7* encodes for a key transcriptional regulator of IFN‐I immune response, and downregulation of *Irf7* was previously demonstrated to be associated with anti‐inflammatory responses.^[^ ^30^ ^]^ Dendritic cells seem to be strongly involved the immune‐microbiota cross‐talk, and may be downregulated in this experiment as shown by decreased signaling of TLR3, a dendritic‐specific receptor involved in dendritic cells maturation and microbiota recognition, in the PTS‐supplemented group of rats (Figure 1C).^[^ ^76^ ^]^ In terms of microbial commensals, RSV and PTS normalize the microbiota composition of mice in various conditions, such as IBD or an experimental model of colitis^[^ ^77^ ^]^ potentially due to their ability to restore intestinal barrier integrity.^[^ ^78^ ^]^

Considering that low‐grade inflammation, i.e., inflammaging, accompanies the process of aging and results in metabolic imbalance and impaired liver functions,^[^ ^55^, ^56^ ^]^ we assessed whether the selected PTS‐target genes are differentially expressed in human liver tissue between young and older individuals (Figure 4), as well as in individuals with liver disease, steatosis or NASH (Figures 5 and 6). Although only *OAS1* demonstrated a statistically significant upregulation with aging, majority of remaining genes showed the same direction of change, although statistically non‐significant (Figure 4). When liver samples from steatosis or NASH patients were analyzed, statistical significance was achieved for majority of genes and in many databases (Figures 5 and 6). The expression patterns for PTS‐target genes in human samples may not fully overlap with expected changes based on the results in our rat model due to several factors, including a low number of participants, a discrepancy between biological and chronological age, and a methodological approach in independent studies. In addition, some PTS‐targets have functions that could be beneficial or harmful depending on the context. For instance, reduction in expression of the *SLC7A5* transporter, and thus in transport of amino acids, mimic a dietary restriction state that is linked to increased longevity.^[^ ^37^, ^39^ ^]^ Hence, PTS reduces *Slc7a5* in blood of healthy rats. However, amino acids play a vital role in liver functions (e.g., glycine), and restricting them in liver disease is not beneficial.^[^ ^79^ ^]^ Hence, we do not observe SLC7A5 increase in databases in patients with liver disease.

In conclusion, our study is the first to characterize transcriptomic patterns in blood of healthy animals consuming diets supplemented with polyphenols. We discover that only PTS, but not RSV or CGA, used at the same molar concentrations, results in detectable modifications in the gene expression landscape. This finding is in concordance with higher bioavailability of PTS that may translate into higher biological activity. PTS incorporation into the diet of healthy rats has shown to target functions associated with hallmarks of aging that are linked together to bring about low‐grade inflammation, i.e., inflammaging, and consequently dysregulation of the immune system and cellular metabolism. Such findings provide a rationale for pre‐clinical and clinical longevity studies and encourage investigations of PTS in decelerating the process of aging and in conditions with chronic inflammation. Since our study was performed in male animals, it will be of interest to explore sex‐dependent effects in future research. Moreover, further research is needed to elucidate whether long‐term consumption is beneficial for complex diseases like cancer, since tumor‐associated immune cells often exhibit aberrantly low levels of inflammation. However, considering known anti‐cancer properties of PTS, it is highly possible that the mechanisms of PTS action depend on the cellular context and differ between healthy and cancer cells. Altogether, our data support the use of PTS in maintaining cellular homeostasis which could prevent disease and support healthy aging.

## 4 Experimental Section

### Animals and Diets

A total of 24 male Fischer 344 rats at 4 weeks of age were obtained from Charles River (Indianapolis, IN, USA) and housed two rats per cage in a temperature‐controlled (24 °C) room with a 12 h light/dark cycle, and ad libitum access to water and a chow diet (i.e., choline‐sufficient amino acid‐defined [CSAA]). After a week of acclimation, rats were randomly divided into four groups, each containing six animals. Using the biological and analytical variability from previous data,^[^ ^80^, ^81^ ^]^ it could be determined that six rats per group (*n* = 6) were required to detect a 50% difference with the variation of the endpoint 25% (i.e., gene expression measured by qRT‐PCR) and with 80% power at a nominal significance of *p* < 0.05. The four groups were as follows: Group 1: fed the CSAA diet; Group 2: fed a CSAA diet supplemented with resveratrol (CSAA+RSV, 1.2 g kg^−1^ of diet); Group 3: fed a CSAA diet supplemented with pterostilbene (CSAA+PTS, 1.34 g kg^−1^ of diet); Group 4: fed a CSAA diet supplemented with chlorogenic acid (CSAA+CGA, 1.9 g kg^−1^ of diet). All diets were pelleted at Dyets Inc. (Bethlehem, PA, USA). The compounds (BIOTANG Inc., Lexington, MA, USA) were used at equal molar concentrations. Following a 20‐day feeding period, blood samples were collected by retro‐orbital plexus sampling from anesthetized rats, where the retro‐orbital plexus was penetrated with a capillary tube to promote blood flow from the capillaries behind the eye. The amount of collected blood did not exceed 10% of circulating blood volume based on body weight. Blood samples were collected from each rat into spray coated K2EDTA tubes (BD & Co., Franklin Lakes, NJ, USA) and stored in −80 °C until RNA was extracted. Rats were treated strictly following the animal use protocol #1112000342 approved by the Institutional Animal Care and Use Committee at the involved institutions.

### Dosage Information

Compounds were incorporated into the diet at concentrations listed above. These concentrations correspond to 86, 96, and 136 mg kg^−1^ of body weight per day for RSV, PTS, and CGA, respectively. The selected doses had been shown to be effective in attenuation of inflammation, metabolic disturbances, and cancer development based on previous studies in animal models. For example, a profound reduction in tumor growth in colon and lung cancers required 50–250 mg of PTS.^[^ ^82^, ^83^ ^]^ RSV at doses of 100–300 mg was effective in reducing liver nodules in rats,^[^ ^84^, ^85^, ^86^ ^]^ and CGA at 100 mg reduced inflammation and oxidative stress preventing myocardial infarction in rats.^[^ ^87^ ^]^ These effective doses were not toxic. Administration of PTS at 30, 300, and 3000 mg kg^−1^ of body weight per day into mice did not produce any toxic effects.^[^ ^88^ ^]^ CGA at a dose of up to 1000 mg kg^−1^ was safe and did not adversely affect metabolism and physiology in rats.^[^ ^89^ ^]^ However, the selected doses were higher compared with what humans can consume per day by eating food sources of PTS, RSV, and CGA. For instance, to ingest 20 mg of PTS or RSV, one would have to eat 1000 or 100 kg of dark‐skinned grapes, respectively.^[^ ^90^, ^91^ ^]^ The dose of CGA in the study corresponded to a consumption of approximately 1550 mg day^−1^ in humans. Considering that on average 800 mg of CGA was in 1 L of coffee, one would have to consume eight cups of coffee per day.^[^ ^92^ ^]^ Thus, the doses used in this studycan be achieved from supplements rather than from a diet, especially for PTS and RSV. Of note, the study cannot exclude that biological effects caused by those higher doses in the animal model were equivalent to those occurring in humans upon habitual consumption of diet rich in polyphenols.

### RNA Extraction from Rat Blood and qRT‐PCR

Mouse RiboPure‐Blood RNA Isolation Kit (Invitrogen) was used to extract RNA from rat whole blood samples as recommended by the manufacturer. Part of total RNA was then processed for cDNA synthesis using 20 U of AMV reverse transcriptase (Roche Diagnostics), according to the manufacturer's protocol. Target genes were amplified in CFX96 Touch Real‐Time PCR Detection System (Bio‐Rad) using 2 µL of cDNA as a template, 400 nM forward and reverse primers (please see Table S1, Supporting Information for sequences), and 10 µL of SsoFast EvaGreen Supermix (Bio‐Rad) in a final volume of 20 µL. The amplification reaction started with denaturation at 95 °C for 10 min, followed by amplification for 60 cycles at 95 °C for 10 s, annealing temperature for 10 s, and 72 °C for 10 s, and ended with a final extension at 72 °C for 10 min. Gene expression was quantified in the CFX Maestro Software (Bio‐Rad) with a standard curve‐based analysis. Quantitative RT‐PCR data were presented as gene of interest/Gapdh (reference gene).

### RNA Sequencing (RNA‐seq)

Total RNA extracted from rat blood samples was processed for RNA‐seq. Quality check was performed using an Agilent Bioanalyzer prior to library preparation from five rats per group, namely, CSAA, CSAA+RSV, CSAA+PTS, and CSAA+CGA. The TruSeq Stranded kit (Illumina, San Diego, CA, USA) was used to prepare libraries followed by sequencing of four rats per lane using the HiSeq2500 on high‐throughput mode (1 × 50 bp reads).

### RNA‐seq Data Pre‐Processing

Raw sequencing reads were pre‐processed by standard quality trimming and adapter removal with Trimmomatic v 0.32^[^ ^93^ ^]^ and FastX‐Toolkit v 0.0.13.2; setting trimscore to 30, trim length of 30, and maximum length set to 51 bp. The final quality score was evaluated with FastQC v 0.11 and FastX. On average, 95% of reads per sample were aligned to the reference genome Rnor version 6.0 using TopHat2 v 2.1^[^ ^94^ ^]^ using the lowest tolerance for mismatches, segment‐mismatch set to 1 instead of 2. Finally, aligned reads were annotated to gene features using the R package Rsubread v 2.8,^[^ ^95^ ^]^ and ENSEMBL IDs were converted to Gene Symbols using biomaRt v 2.46.^[^ ^96^ ^]^ Genes with fewer than two reads in five or more samples were classified as lowly expressed and removed from the analysis. Lastly, expression values for genes with duplicated ENSEMBL IDs or Gene Symbols were averaged, resulting in a gene expression table with 16067 genes. RNA sequencing raw reads and count data were available in Gene Expression Omnibus (GEO, accession number: GSE278528).

### Differential Expression Analysis Workflow

The study used DESeq2 v 1.30^[^ ^97^ ^]^ for all differential expression (DE) analysis with default parameters. The study used two different experimental designs *∼ diet* for regular DE, and *∼ diet + TCell + Bcell + Macrophage* to incorporate cell scores determined by CIBERSORTx.^[^ ^98^ ^]^ Note that the neutrophils fraction was not included in the design since it was linearly dependent on all other cell fractions. The study defined differentially expressed genes (DEGs) using an FDR threshold less or equal to 0.05 (FDR ≤ 0.05), and an absolute fold change greater than 2 (|log2(FC)| > 1).

### Ortholog‐Guided Cell Type Deconvolution

The study used the CIBERSORTx^[^ ^98^ ^]^ online platform for estimating cellular fractions from the whole blood samples (now referenced as mixture data); the study targeted the deconvolution of T‐cells, B‐cells, macrophages, and neutrophils. Standard deconvolution workflow first required the definition of a weight matrix. This matrix captured the importance of every gene in the reference data to identify each one of the targeted cell types. The study used as reference *Mus musculus* sorted blood dataset from the Haemosphere database^[^ ^22^ ^]^ which covered 47 643 unique ENSEMBL gene IDs and was composed of 15 B‐cell samples, 11 Macrophage samples, 6 Neutrophil samples, and 16 T‐cell samples. Due to the lack of a similar reference for *Rattus norvegicus*, the study reduced reference and mixture datasets to a common set of 9096 orthologs classified as “one‐to‐one orthologs” and “high confidence” by ENSEMBL using biomaRt. These reference blood samples were used as input in CIBERSORTx to estimate the signature matrix with default parameters. The signature matrix was subsequently used to estimate cell fractions using 1000 permutations in the absolute mode of CIBERSORTx.

### Gene Set Enrichment Analysis

Gene set enrichment analysis (GSEA) was performed using the fast gene set enrichment analysis R package (fgsea)^[^ ^99^ ^]^ and the PANTHER platform v 19.0.^[^ ^23^ ^]^ The fgsea package v 1.27 was used to identify enriched pathways from the Reactome Pathway Database^[^ ^100^ ^]^ accessed through the package reactome.db v 1.82. The study used biomaRt v 2.54 to remove genes without an associated Entrez Gene ID since the reactome.db package only contained Entrez Gene IDs. As recommended by fgsea authors, the study ranked the list of genes based on the test statistic calculated during the differential expression analysis by DESeq2. Only pathways with an adjusted *p*‐value ≤ 0.05 were considered. Benjamini and Hochberg (BH) false discovery rate (FDR) method was applied as a *p*‐value adjustment.

### Analysis of Publicly Available Datasets

The following search terms were applied for RNA‐seq data in the Gene Expression Omnibus (GEO) for human whole blood and liver tissue: “young,” “elderly,” “aging,” “blood,” “whole blood,” “liver,” “steatosis,” “NAFL,” “NAFLD,” “NASH.” Identified datasets were listed in Tables S3, S4, Supporting Information along with corresponding cohort characteristics.

Expression data from publicly available datasets were presented in boxplots and depicted minimum, interquartile range (IQR), and maximum. GEO2R (http://www.ncbi.nlm.nih.gov/geo/geo2r/) was applied to determine the difference in mRNA expression of selected genes between young and aged individuals or between healthy individuals and patients with liver disease in available GEO series. GEO2R was an interactive web tool for comparing two groups of data to analyze any GEO series.^[^ ^101^ ^]^ Statistical analyses on the data from GEO2R that followed a normal distribution, as evaluated by Shapiro‐Wilk test, were performed using Mann–Whitney *U* test for sets with two groups and Tukey's test for sets with more than two groups. For not normally distributed data (i.e., PLTP in GSE33814 and OAS1 in GSE89632), Kruskal–Wallis test was applied. Benjamini and Hochberg (BH) false discovery rate (FDR) method was applied as a *p*‐value adjustment.

### Statistical Analysis

Raw RNA‐seq data were analyzed as described in the Results section. Statistical analysis of qRT‐PCR was performed using Mann–Whitney *U* test. Each value represented the mean ± S.D. of *n* = 6 rats per group. The results were considered statistically significant when *p* < 0.05.

## Conflict of Interest

The authors declare no conflict of interest.

## Supporting information



Supporting Information

Supporting Information

Supporting Information

Supporting Information

Supporting Information

## Data Availability

RNA sequencing data available in the Gene Expression Omnibus (GEO, accession number: GSE278528).
