# Supplementary material for: Pterostilbene Targets Hallmarks of Aging in the Gene Expression Landscape in Blood of Healthy Rats
Source: Mol Nutr Food Res. 2024 Nov 19;68(24):2400662. doi: 10.1002/mnfr.202400662 (PMC11670294; doi:10.1002/mnfr.202400662)

**Supplementary Figure S1. Exploratory data analysis for all diet groups.** (A) Density plot for variance stabilized transcripts computed with DESeq2 to identify differences between samples not due to gene size variability. We identified sample RSV_14 to be an outlier and it was removed for downstream analyses. (B) Principal component analysis shows a clearer separation between CSAA reference diet (blue) and PTS supplemented diet (red), compared to other diet groups. (C) PCA analysis restricted to CSAA and PTS samples, the combination of PC2 and PC3 clustered samples in the CSAA and PTS groups.


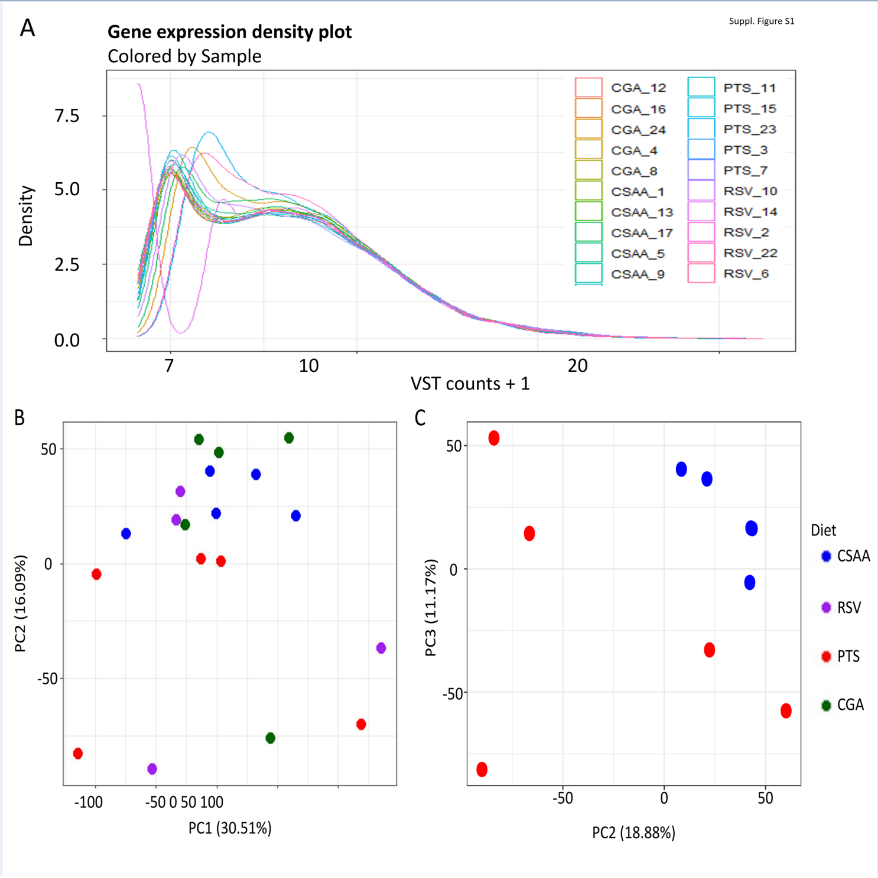


**Supplementary Figure S2. Standard differential expression analysis.** (A) P-value histogram for comparison CSAA diet vs PTS diet. (B) Volcano plot identifying four differential expressed genes, red dots, that met the criteria of FDR<=0.05 and |log2(FC)| > 1. Blue dots represent genes with a statistically significant change in expression that did not meet the fold change criteria.


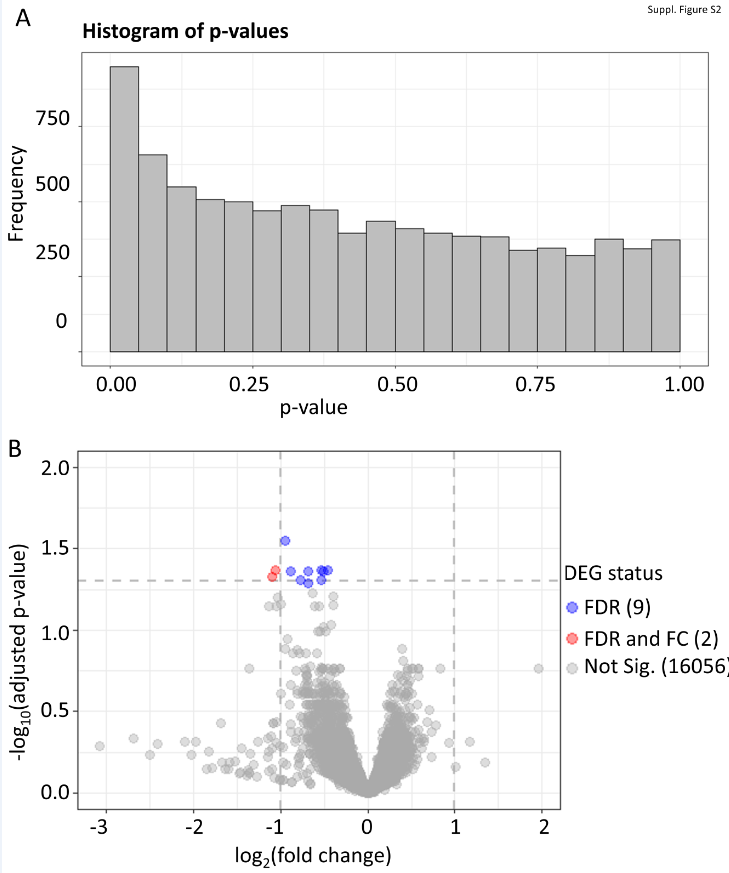


**Supplementary Figure S3. CIBERSORTx results visualization**. (A) Estimated cell-type fractions across all samples. (B) Weight of every gene in the matrix weights, highlighted in red the corresponding values for differentially expressed genes identified by the ortholog deconvolution pipeline. From left to right, values correspond to Neb, P2rx1, Alad, Cd63, Pdlim1, Parp12, Oasl, Hpse, Klhl2, Clic4, Pld4, and Abtb1.


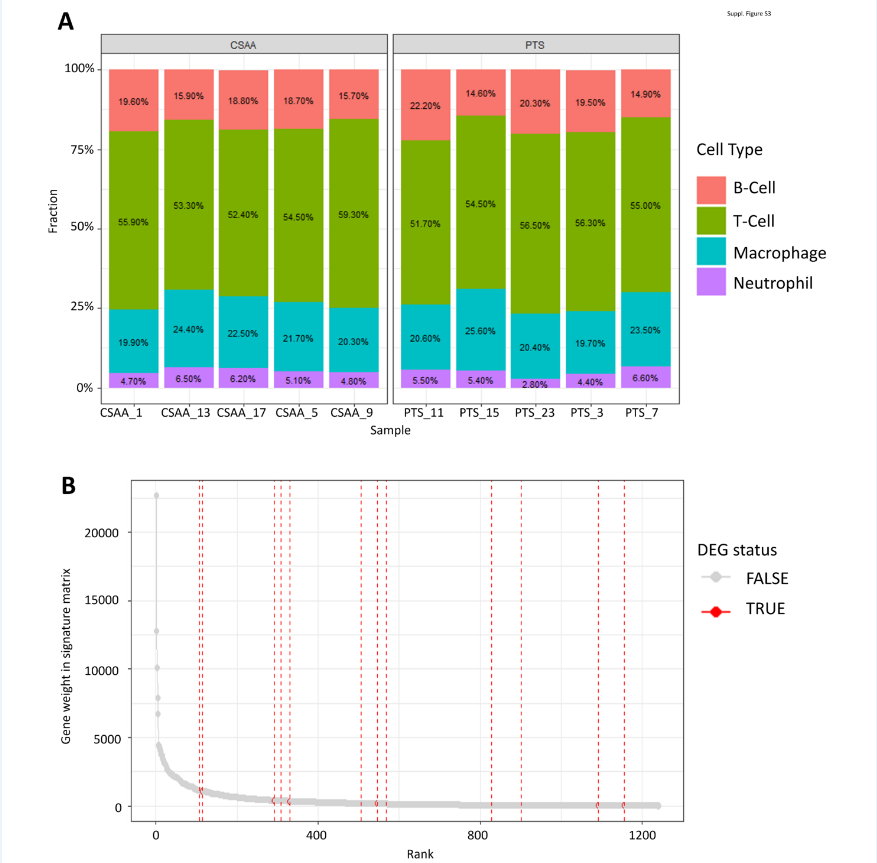

Supplement: Supplementary file 1 — Supporting Information [file MNFR-68-2400662-s002.docx]
