# Supplementary material for: Pterostilbene Targets Hallmarks of Aging in the Gene Expression Landscape in Blood of Healthy Rats
Source: Mol Nutr Food Res. 2024 Nov 19;68(24):2400662. doi: 10.1002/mnfr.202400662 (PMC11670294; doi:10.1002/mnfr.202400662)
Supplement: Supplementary file 2 — Supporting Information [file MNFR-68-2400662-s003.docx]

**Supplementary Table S1.** Primer sequences used in gene expression analysis by qRT-PCR,

| **Table S1**. Primer sequences used in qRT-PCR analysis. | | | |
| --- | --- | --- | --- |
| **Gene** | **Primer sequences (qRT-PCR)** | **Annealing temperature [°C]** | **Amplicon length [bp]** |
| Gapdh RAT | FW 5'-TCTCTGCTCCTCCCTGTTCT-3' RV 5'-TACGGCCAAATCCGTTCACA-3' | 59 | 104 |
| Irf7  RAT | FW 5’-TCAACACCCAGTTCTGATGAC-3’  RV 5’-CTATACAGGAACACGCATCTGG-3’ | 59 | 120 |
| Oas2  RAT | FW 5’-CGATGTGCTGAAAGTGGTTAAG-3’  RV 5’-TTTGAAGAGACCGGTGAAGAG-3’ | 59 | 100 |
| Oas1a  RAT | FW 5’-AGGAGAGATGCTTCCGAGATA-3’  RV 5’-TGACTTGCCCTTGAGTGTG-3’ | 59 | 98 |
| Ifi27  RAT | FW 5’-CAAGACTGCTGTCGCCAT-3’  RV 5’-CAATGCCTGACCCAGTGAA-3’ | 59 | 92 |
| Lgals3bp  RAT | FW 5’-GGGCAGCAGTGTCATCAT-3’  RV 5’-ACCTCGATTCTTCGGGAGTA -3’ | 59 | 90 |
| Rtp4  RAT | FW 5’-GACCCTGCACTTGGATAAGAA-3’  RV 5’-ACATCTGGAACACTGGAACC-3’ | 59 | 100 |
| Slc7a5  RAT | FW 5’-CGCCACATACCTGCTCAA-3’  RV 5’-GTTCACAGCCGTGAGTAGTAG-3’ | 59 | 103 |
| Pltp  RAT | FW 5’-TGAACTTGTGGGCATCGATTA-3’  RV 5’-CTCCTTGAGAGGGAAGAATGC-3’ | 59 | 100 |
